# Supplementary material for: Addressing the mean-correlation relationship in co-expression analysis
Source: PLoS Comput Biol. 2022 Mar 30;18(3):e1009954. doi: 10.1371/journal.pcbi.1009954 (PMC9009771; doi:10.1371/journal.pcbi.1009954)
Supplement: S1 Text — Fig A: Variance stabilization does not remove the mean-correlation relationship. Same raw data as Figs 2 and 3, but we apply a variance stabilizing transformation (as implemented by DESeq2) followed by removing 4 principal components. (a) Like Fig 3a, i.e. densities of the Pearson correlation between all genes within each of 10 expression bins (background) as well as the top 0.1%. (b) Like Fig 2c, i.e. the relationship between IQR of gene-gene correlation distribution and the lowest of the two expression bins associated with the submatrix. Fig B: Distribution comparison for different submatrices of the observed correlation matrix (after removing the top 4 PCs). Same data as Fig 2. Quantile-quantile plots comparing the distribution of Pearson correlations in various (i, i) submatrices (y-axis) to the (9, 9) submatrix(x-axis). Fig C: The background and signal components depends on expression level across many tissues. Data is 9 different GTEx tissues, all with 4 PCs removed. Distributions of Pearson correlations for genes within each expression bin, supplemented with the distribution of the top 0.1% of correlations (within each expression bin). Fig D: The background and signal components does not depend on expression level after spatial quantile normalization. Data is 9 different GTEx tissues, all with 4 PCs removed. Like Fig C but after applying spatial quantile normalization. Fig E: The impact of SpQN on transcription factor co-expression, all transcription factors. Like Fig 7a. The percent increase in the number of edges (y-axis) identified after thresholding (x-axis) the correlation matrix, for edges involving transcription factors. Fig F: The impact of SpQN on transcription factor co-expression, PPI transcription factors. Like Fig 7b. The percent increase in the number of edges (y-axis) identified after thresholding (x-axis) the correlation matrix, for edges between genes with protein-protein interactions where one of the involved genes is a transcription factor. [file pcbi.1009954.s001.pdf]

SUPPORTING INFORMATION – S1 TEXT

Addressing the mean-correlation relationship in co-expression analysis

Yi Wang, Stephanie C. Hicks, Kasper D. Hansen*

*Correspondence to khansen@jhsph.edu

Contents

1. Table A.
2. Figures A-N.

SUPPLEMENTAL TABLES

Table A. Number of principal components to be removed, as estimated using SVA.

Tissue name	Number of PCs removed
Adipose Subcutaneous	30
Adrenal Gland	20
Artery Tibial	27
Brain Cerebellum	15
Brain Cortex	12
Breast Mammary	14
Colon Transverse	10
Esophagus Mucosa	24
Heart Left Ventricle	16

SUPPLEMENTAL FIGURES

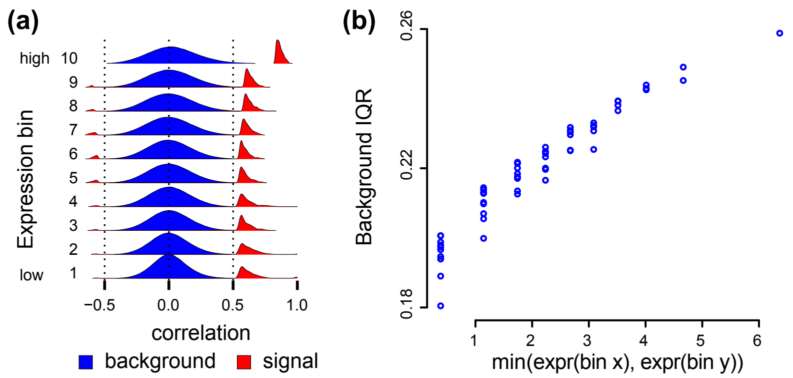

Fig A. Variance stabilization does not remove the mean-correlation relationship. Same raw data as Figs 2 and 3, but we apply a variance stabilizing transformation (as implemented by DESeq2) followed by removing 4 principal components. **(a)** Like Fig 3a, i.e. densities of the Pearson correlation between all genes within each of 10 expression bins (background) as well as the top 0.1%. **(b)** Like Fig 2c, i.e. the relationship between IQR of gene-gene correlation distribution and the lowest of the two expression bins associated with the submatrix.

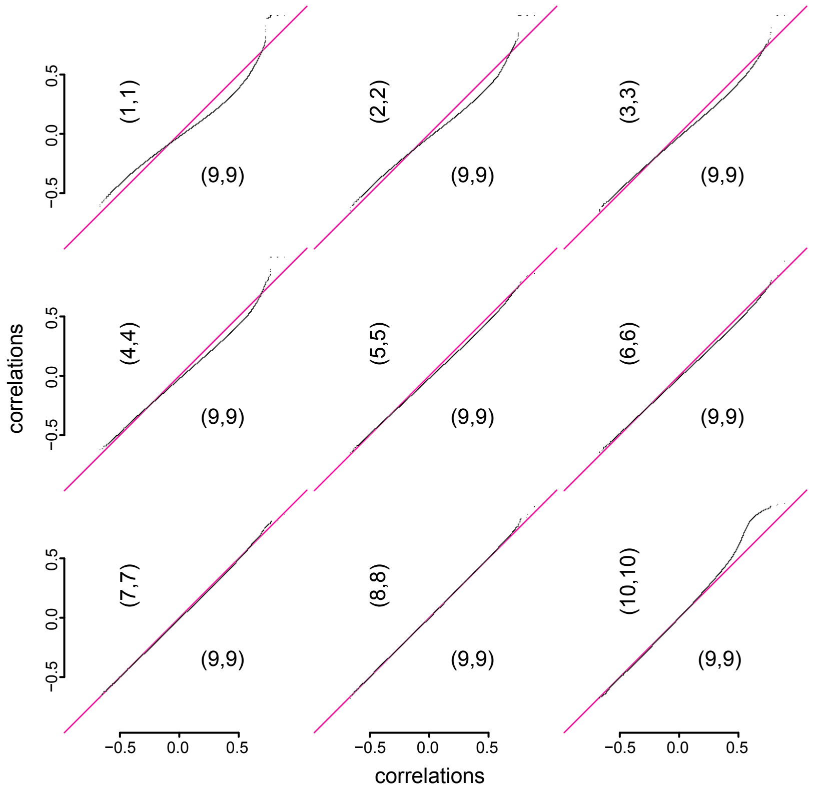

Fig B. Distribution comparison for different submatrices of the observed correlation matrix (after removing the top 4 PCs). Same data as Figure 2. Quantile-quantile plots comparing the distribution of Pearson correlations in various (i, i) submatrices (y-axis) to the $(9, 9)$ submatrix (x-axis).

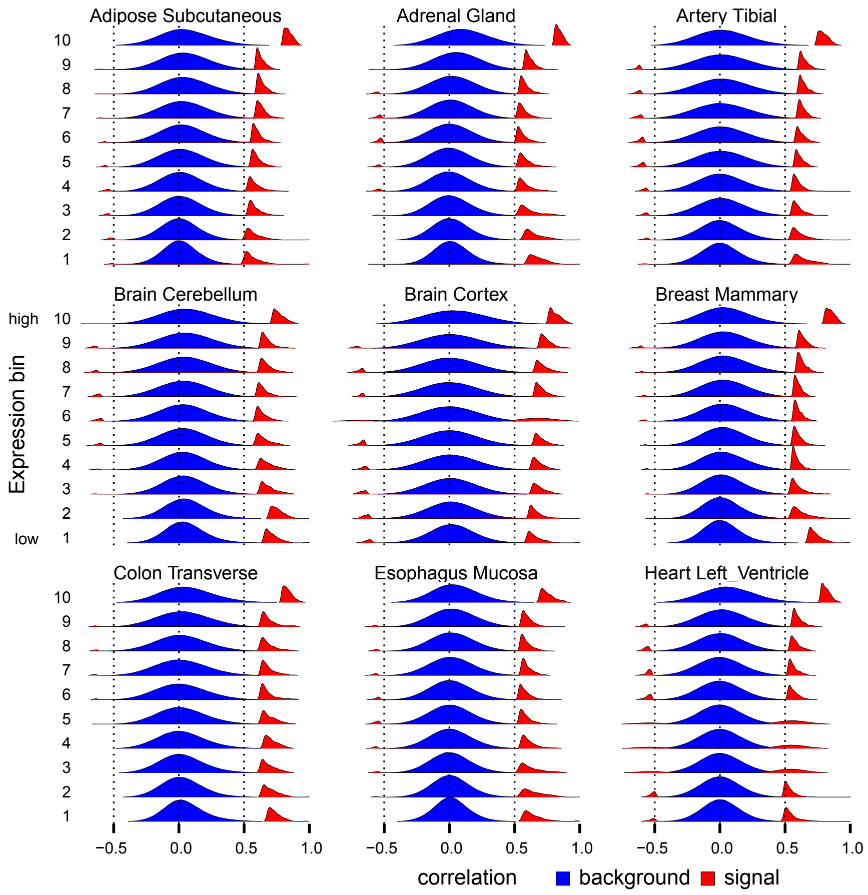

Fig C. The background and signal components depends on expression level across many tissues. Data is 9 different GTEx tissues, all with 4 PCs removed. Distributions of Pearson correlations for genes within each expression bin, supplemented with the distribution of the top 0.1 % of correlations (within each expression bin).

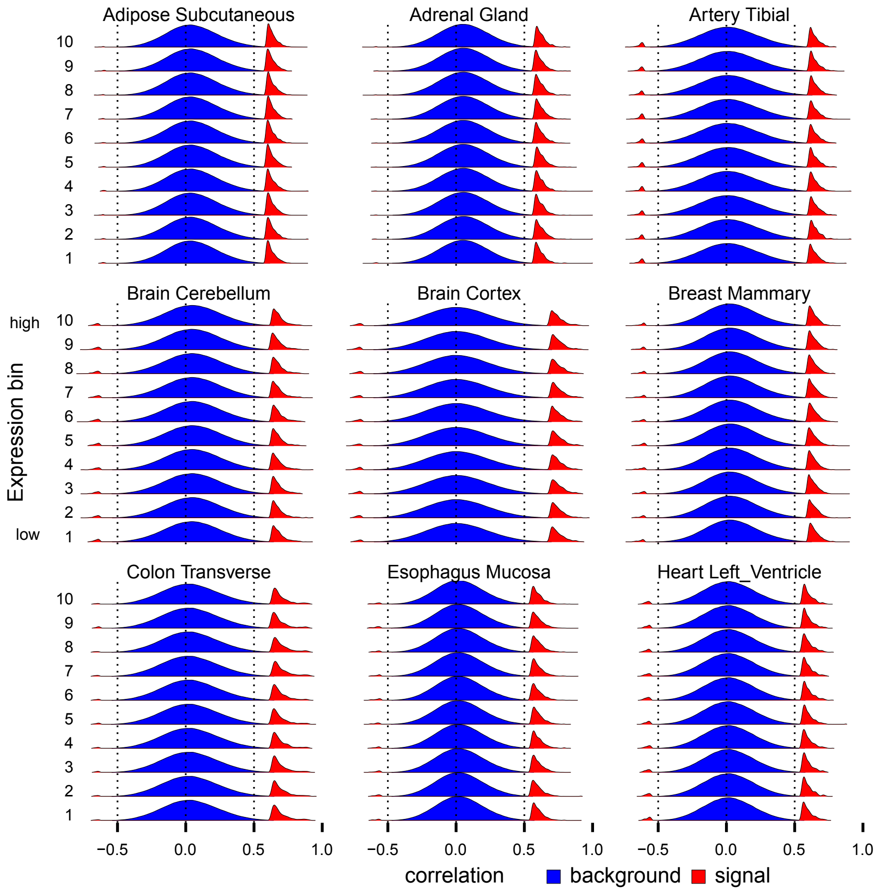

Fig D. The background and signal components does not depend on expression level after spatial quantile normalization. Data is 9 different GTEx tissues, all with 4 PCs removed. Like Fig C but after applying spatial quantile normalization.

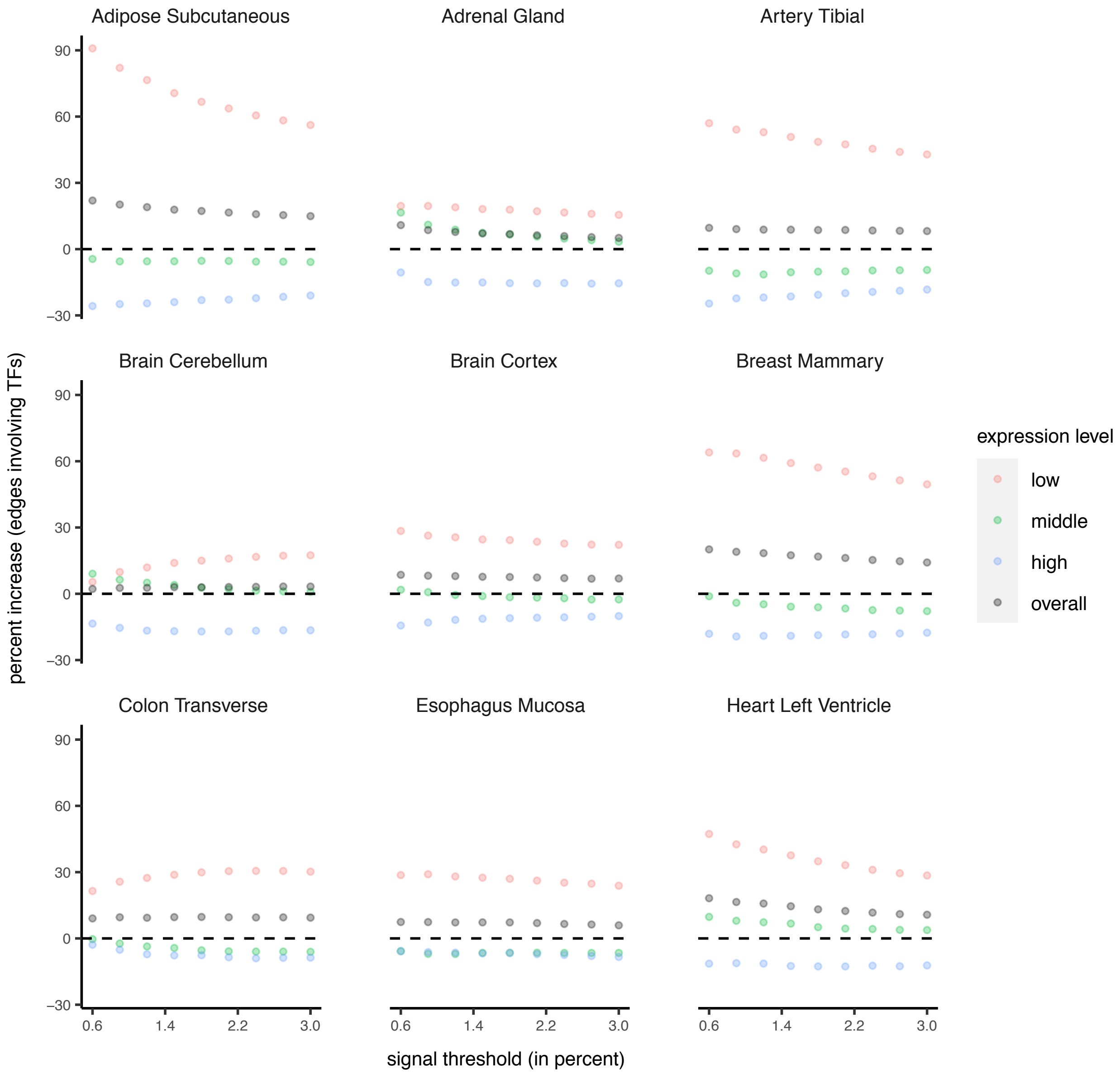

Fig E. The impact of SpQN on transcription factor co-expression, all transcription factors. Like Fig 7a. The percent increase in the number of edges (y-axis) identified after thresholding (x-axis) the correlation matrix, for edges involving transcription factors.

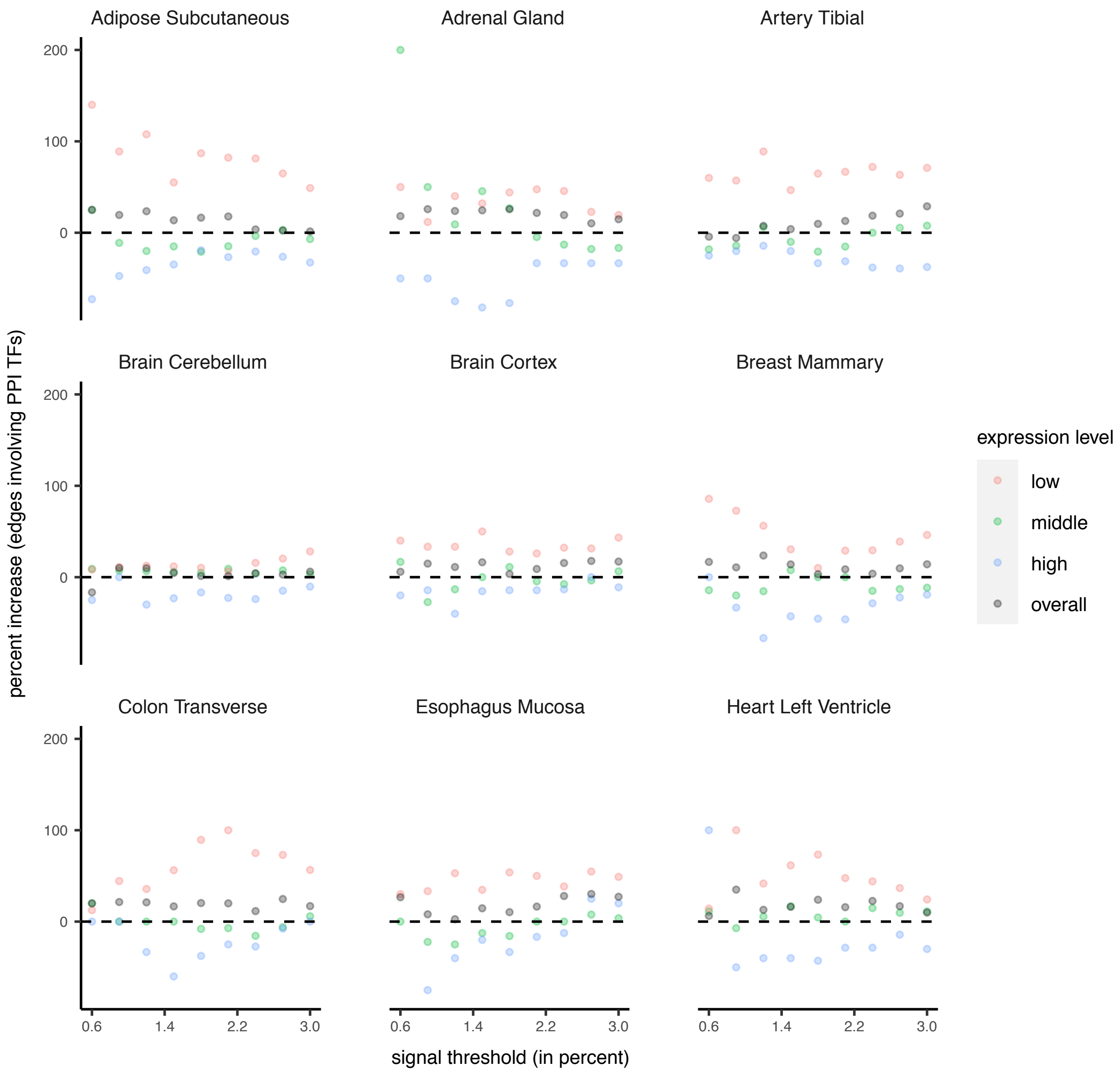

Fig F. The impact of SpQN on transcription factor co-expression, PPI transcription factors. Like Fig 7b. The percent increase in the number of edges (y-axis) identified after thresholding (x-axis) the correlation matrix, for edges between genes with protein-protein interactions where one of the involved genes is a transcription factor.

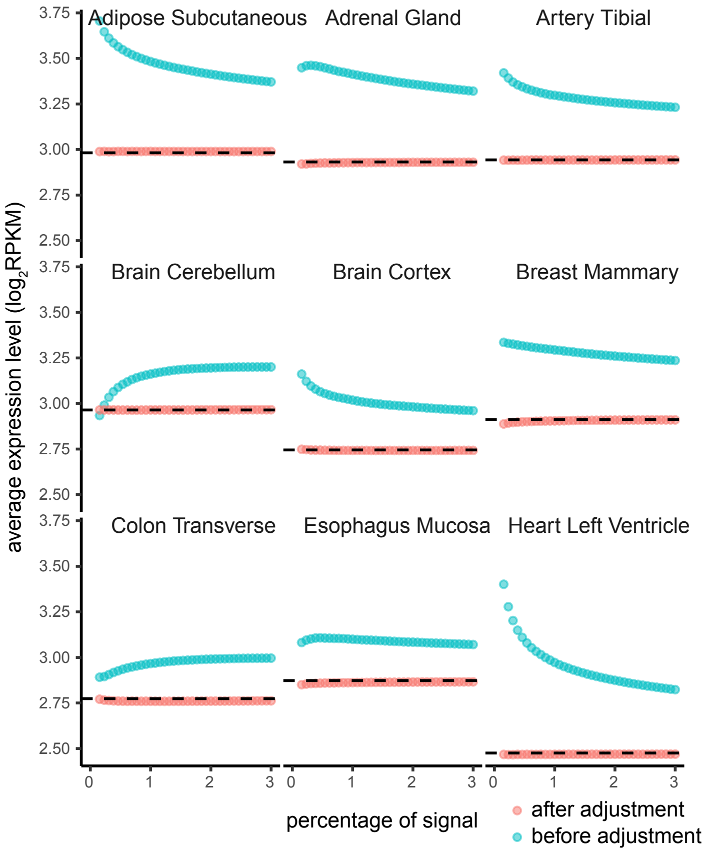

Fig G. The relationship between the signal threshold (in percentage) and the expression level (before and after SpQN adjustment). We define the co-expression signal threshold (x-axis) as the top percentage of absolute correlation values (ranging between 0 and 3%). For a given signal threshold, we calculate the average expression level for each tissue (y-axis). We compare the expression levels before (blue) and after SpQN adjustment (pink). The average gene expression level for each tissue is shown by the dotted black line.

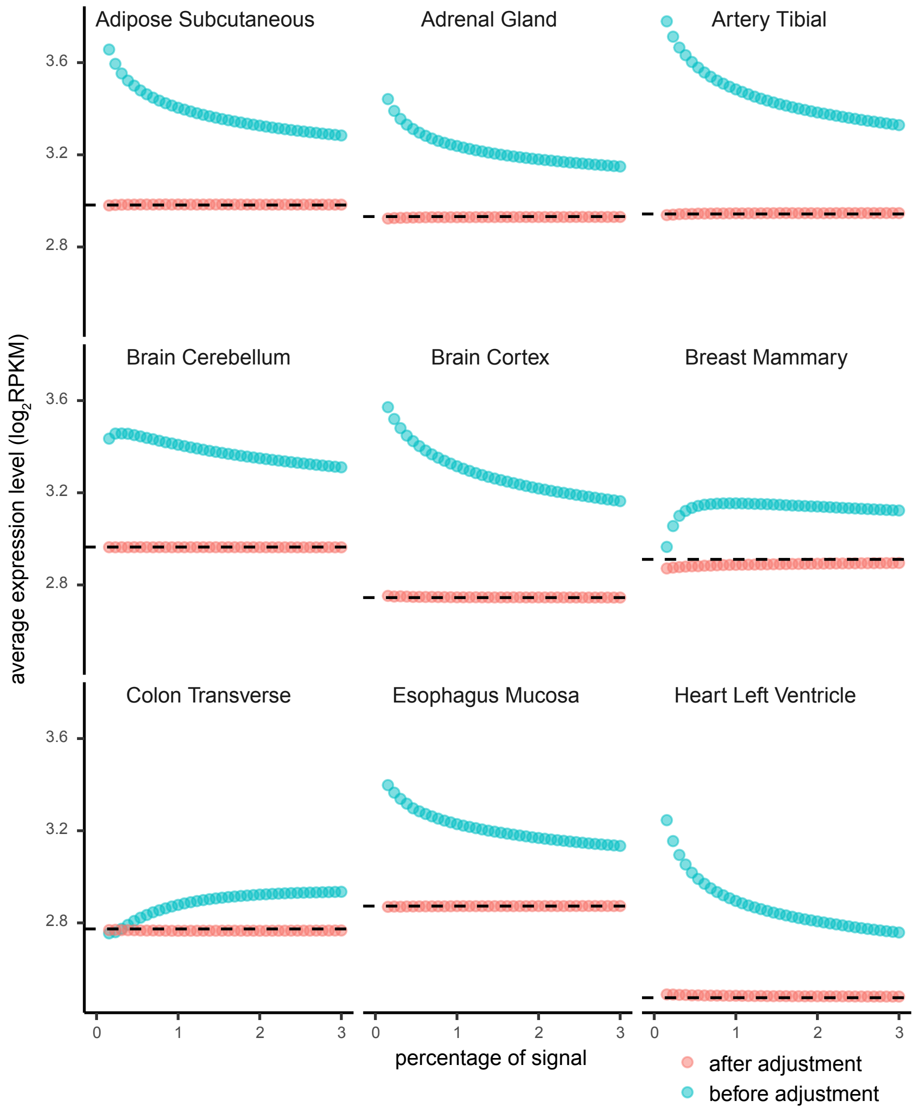

Fig H. The relationship between the percentage of co-expression signals and the expression bias. Like FigG, but where SVA was used to decide the number of PCs to be removed in the correlation matrix

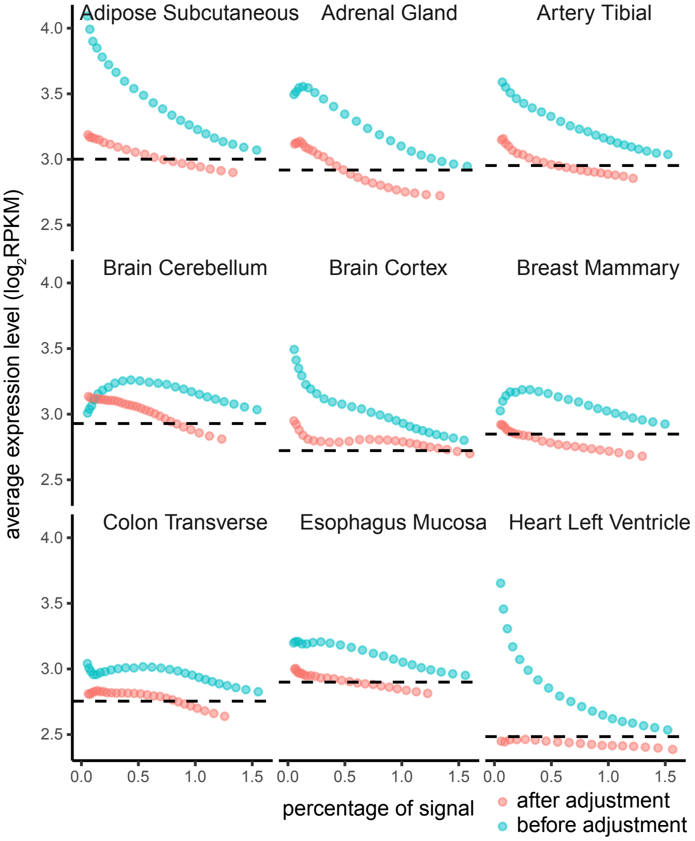

Fig I. The expression bias in graphical lasso network inference. The expression levels of networks inferred by graphical lasso. Different values of the tuning parameter (ρ) results in different network sizes (x-axis) with higher values of the tuning parameter leading to smaller networks. The average gene expression for each tissue is shown by the dotted black line.

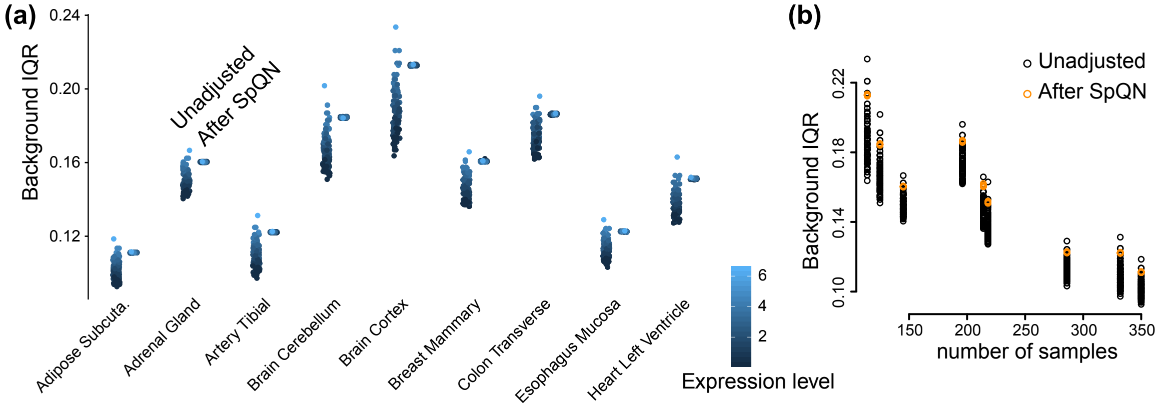

Fig J. IQR of Pearson correlations in each bin for 9 GTEx tissues (before and after SpQN adjustment). Bulk RNA-seq data from **GTEx2017** from 9 tissues. Each tissue has a number of PCs removed based on the estimate from SVA, as suggested by **Parsana2019**. **(a)** Background IQR for unadjusted (left smear) and SpQN-adjusted (right smear) gene-gene correlation distributions for all expression bins across 9 GTEx tissues. Color indicates expression level. **(b)** The relationship between sample size (x-axis) and IQR for correlations (y-axis) before and after adjustment.

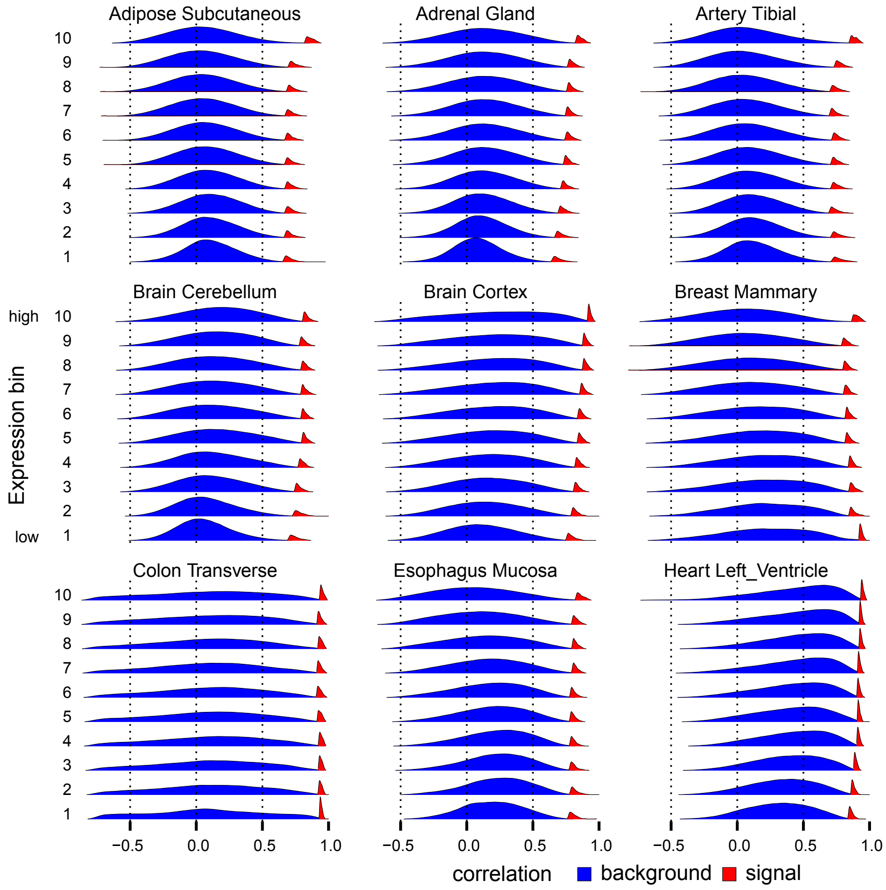

Fig K. The background and signal components depend on expression level (before removing top PCs). Distributions of Pearson correlations for background genes (within each expression bin), supplemented with the distribution of the top 0.1 % of correlations (within each expression bin).

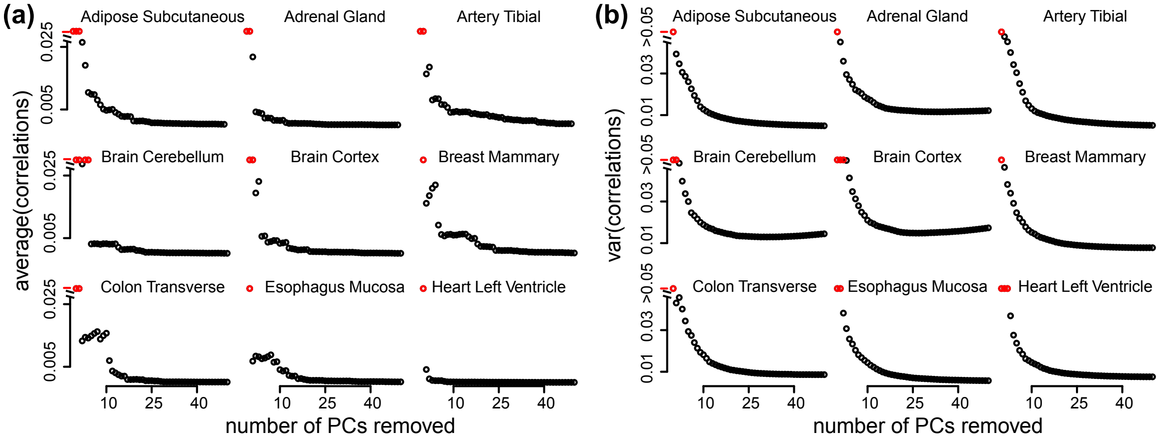

Fig L. The effect of removing principal components on bias and variance of the background distribution. (a) Average bias, defined as the average of the median of the 10 background distributions. **(b)** Average variance, defined as the average variance of the 10 background distributions. Red colored points have bias or variance exceeding the limits of the plot.

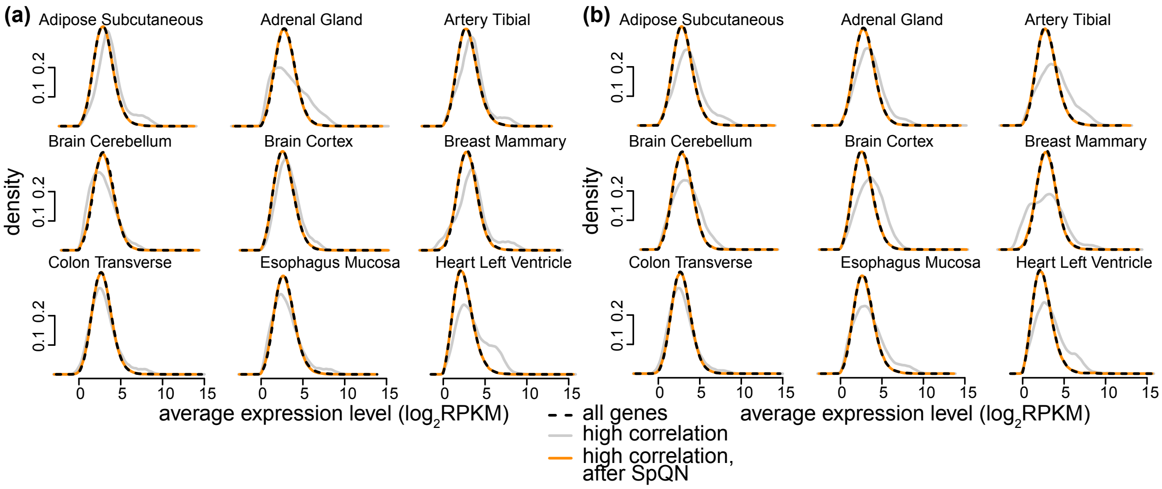

Fig M. The effect of removing principal components (PCs) on bias towards highly expressed genes. As Fig 5d but for 9 tissues and two different approaches for removing PCs. **(a)** 4 PCs were removed from the correlation matrix. **(b)** SVA was used to estimate the number of PCs to remove (range: 10-30 PCs).

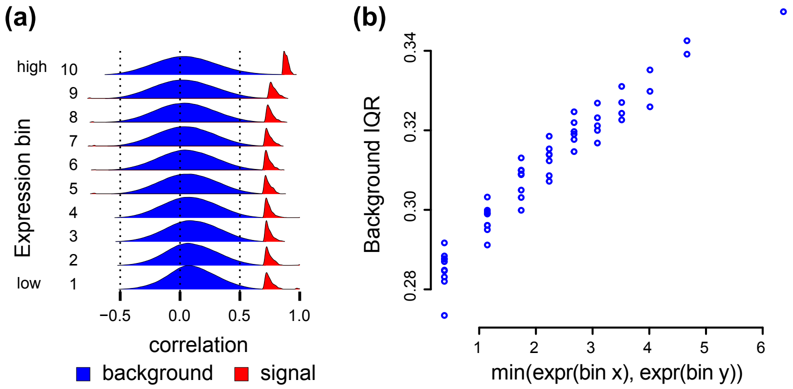

Fig N. ComBat does not remove the mean-correlation relationship. Same raw data as Figs 2 and 3. We apply ComBat (with the date of nucleic acid isolation as batch variable) prior to constructing the expression matrix, instead of removing principal components. **(a)** Like Fig 3a, i.e. densities of the Pearson correlation between all genes within each of 10 expression bins (background) as well as the top 0.1. **(b)** Like Fig 2c, i.e. the relationship between IQR of gene-gene correlation distribution and the lowest of the two expression bins associated with the submatrix.
