# Supplementary material for: Addressing the mean-correlation relationship in co-expression analysis
Source: PLoS Comput Biol. 2022 Mar 30;18(3):e1009954. doi: 10.1371/journal.pcbi.1009954 (PMC9009771; doi:10.1371/journal.pcbi.1009954)
Supplement: Attachment — Submitted filename: response.pdf [file pcbi.1009954.s003.pdf]

Addressing the mean-correlation relationship in co-expression analysis
PCOMPBIOL-D-21-00859

Response to reviews

Yi Wang

Stephanie C. Hicks

Kasper D. Hansen

Editor

(We have submitted a track changes document relative to the typesetting of our first submission. Additional text are marked with red. Deleted text are not marked.)

> "One issue is that while the question for how to best exploit co-expression for biological discovery continues to be an important area of research, some indication of whether the approach is actually an improvement when it comes to biological inference. The reviewer acknowledges that this may be difficult to convincingly address, but at least needs to be acknowledged by the authors and discussed as a limitation. A second important point is the need for focus on comparisons to other potential approaches. The approach proposed is interesting and likely to help remove bias in gene correlation studies."

Response: Thank you. We have addressed the reviewers comments below.

> "There is a third issue that the reviewers do not mention but I believe is important to consider. In addition to mean expression level which you convincingly demonstrate is a driver of gene correlations, another driver of strong gene correlations in building co-expression networks is the statistical leverage of high differentials in expression occurring in genes across anatomic regions, or cells. Whereas the mean correlation will of course be raised by strong differentials, this mean by only be raised modestly overall. This is particularly important as most studies begin with a selection of higher differentially expression genes. It would be interesting to understand this effect and its relationship to or refinement of your model. Based on this feedback, I would like to invite you to submit a revised version of your manuscript addressing these considerations."

Response: The interpretation of a co-expression analysis will depend on what is being correlated. In our manuscript, we focus on what we call the classic co-expression setup, where correlation is computed between biological replicates. Such correlations should reflect differences in genetics and environment between the different samples. The editor suggests considering an alternative setting where correlations are computed across samples where we expect strong differential expression – for example across anatomical regions, tissues, or cell types. While different from a "classic" analysis, this is a type of correlation analysis appears to be increasingly used in the literature. As the editor notes, it is not at all clear that such a correlation matrix has a mean-correlation relationship as we define it.

To investigate this, we first note that the scRNA-seq dataset we use in our manuscript (and which exhibits a mean-correlation relationship) indeed contains multiple cell types and is therefore an example of a correlation matrix computed in a differential setting. To further expand on the editor's question, we have performed two additional analyses: one in GTEx and one on a drosophila embryonic development time course.

For GTEx, we selected 100 samples from each of 3 tissues, to form an aggregate set of 300 samples. This is a sample size which is comparable to the GTEx tissue sample size we use. We then performed our usual assessments of the mean-correlation relationship. Perhaps unexpected, this reveals a mean-correlation relationship similar to what we see between replicates within a tissue (Figure 1a,b).

We have also tried to look at a time course experiment in *drosophila melanogaster*. This experiment is a developmental time course experiment in the fruitfly embyro and consists of 30 samples; far less than the GTEx data we otherwise use. This also shows a mean-correlation relationship (Figure 1c,d) although the differences in IQR are smaller than we usually see (so the relationship is not as strong). We also observe that (a) the background distributions are not centered and (b) they are much wider than for GTEx (c) there are outliers in Figure 1d. Based on our analysis in the paper, we interpret this to mean that the sample size is small and there is still residual unwanted variation. Nevertheless, it suggests the presence of a mean-correlation relationship.

Perhaps the editor was intending to *only* do co-expression amongst differentially expressed genes. That is a different question and we believe it violates the assumption of our method. To be specific, we are utilizing that *most* gene-gene correlations are background (ie. a true correlation of zero). This would not be true for a correlation matrix computed only on differentially expressed genes.

To reflect these analyses, we have created a new section in our manuscript, titled "Co-expression in a differential setting" (last section in Results), where we write

" *So far, we have examined correlation matrices obtained from considering biological replicates within a condition; we consider this the classic co-expression setting. An alternative is to compute correlation matrices where samples are associated with different conditions (including cell types or tissues). We call this the differential setting and this yields a different interpretation of the resulting correlation matrix. An important question is whether such a correlation matrix exhibits a mean-correlation relationship. The answer is not straightforward, because two genes which are both differentially expressed, will be highly correlated, but each gene may be lowly expressed in one condition and highly expressed in another condition.*

To examine this question, we consider two scenarios. First, we create a dataset by randomly sampling 100 individuals from each of 3 tissues for a total of 300 samples, a sample size similar to the GTEx tissues previously considered. This pooled dataset exhibits the same mean-correlation relationship as other datasets we have considered (Figure 12a,b). Next, we consider data from a time course experiment in the developing drosophila embyro, with a total of 30 samples (substantially smaller than other datasets we have considered). This matrix also exhibits a mean-correlation relationship (Figure 12c,d), although we make three observations (1) there is substantial variation between the background IQR of different bins associated with the same expression level and (2) the observed background IQRs are substantially larger than observed elsewhere and therefore (3) the change in background IQRs relative to their variation is smaller then for other datasets we have considered. We hypothesize these observations are the result of the substantially smaller sample

size in this dataset.

These two examples show that co-expression analysis in a differential setting may exhibit a mean-correlation relationship. How often this is true, is an open question, but it is easy to assess as part of any co-expression analysis."

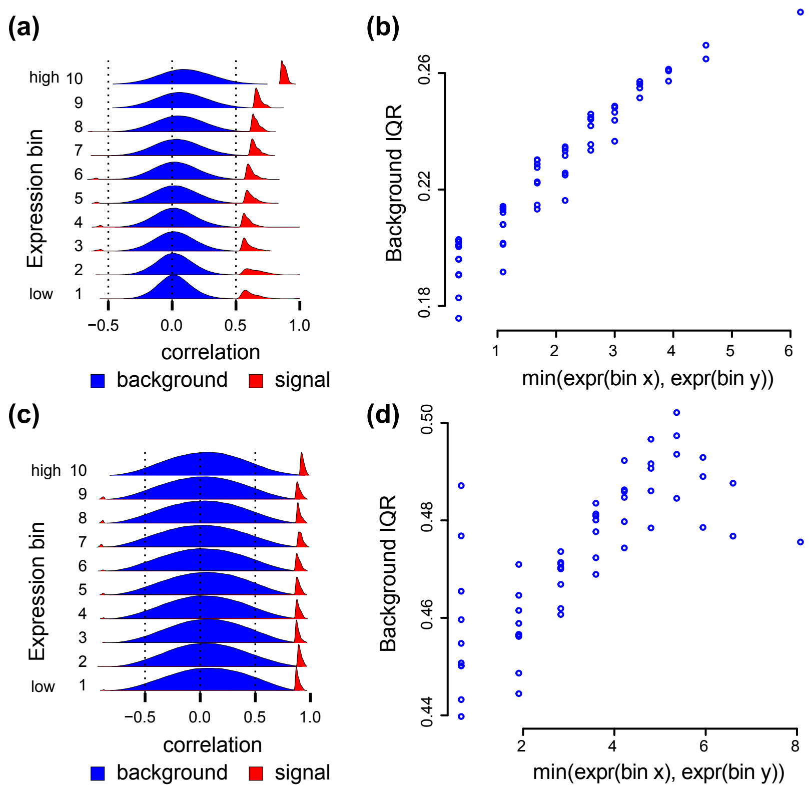

Figure 1. Mean-correlation in a differential setting. Data in (a,b): 100 samples were randomly selected from each of 3 GTEx tissues (adipose subcutaneous, adrenal gland and artery tibial) for a total of 300 samples. We removed 4 principal components from the resulting correlation matrix. Data in (c,d): bulk RNA-seq of a time course experiment on drosophila embryonic development with 30 samples. We removed 5 principal components from the resulting correlation matrix. **(a)** Densities of the Pearson correlation between gene pairs stratified by overall expression, for the GTEx data. **(b)** The relationship between IQRs of the Pearson correlations between all genes in a submatrix (y-axis), and the minimum between the average expression level of the two bins associated with the submatrix (x-axis), for the GTEx data. **(c)** Like (a), but for the drosophila data. **(d)** Like (b), but for the drosophila data.

> "We cannot make any decision about publication until we have seen the revised manuscript and your response to the reviewers' comments. Your revised manuscript is also likely to be sent to reviewers for further evaluation.
>
> When you are ready to resubmit, please upload the following:

[1] A letter containing a detailed list of your responses to the review comments and a description of the changes you have made in the manuscript. Please note while forming your response, if your article is accepted, you may have the opportunity to make the peer review history publicly available. The record will include editor decision letters (with reviews) and your responses to reviewer comments. If eligible, we will contact you to opt in or out.

[2] Two versions of the revised manuscript: one with either highlights or tracked changes denoting where the text has been changed; the other a clean version (uploaded as the manuscript file)."

Reviewer 1

(We have submitted a track changes document relative to the typesetting of our first submission. Additional text are marked with red. Deleted text are not marked.)

> "Overview: The authors convincingly demonstrate a dependence between gene-gene correlations and gene expression. They propose a method to correct for this dependence and show that after applying this method, the dependence between correlation and expression is greatly reduced, perhaps even removed. As the authors note in the Discussion, this work may be applicable to much wider range of scenarios than those described in this work. My primary concerns focus on a lack of comparisons to other potential approaches. I have a few other comments and suggestions that I describe in detail below."

Response: Thank you.

> "Major Comments:
>
> 1. It is difficult to fully appreciate the performance of the SpQN method without comparing it to another approach. While I appreciate that there aren't (to my knowledge) direct competitors, I would encourage the authors to consider whether there are other approaches that could serve as a comparison. For example, would applying a variance-stabilizing transformation largely eliminate the need for SpQN?"

Response: To address this, we applied a variance stabilizing transformation as implemented in the DESeq2 package (function `varianceStabilizingTransformation`). We follow this by removing 4 PCs. Figure 2 shows that this approach does not remove the observed mean-correlation relationship although it decreases the effect slightly. While it may be possible that we need to remove another number of principal components to remove batch effects, this nevertheless demonstrates that the mean-correlation relationship does not get removed by stabilizing the variances.

We now write, in the Results section "The distribution of gene-gene correlations depends on gene expression level":

" *The mean-correlation relationship is still present in data processed with a variance stabilizing transformation, a transformation which aims at removing the known mean-variance relationship in RNA-seq data (Supplemental Figure S1).*"

> "2. While this is becoming common practice in the field, regressing out the top N PCs likely removes substantial biological variation in addition to the technical variation they are assumed to represent. In the case of the GTEx data, for which RNA extraction and RNA sequencing dates are available, it would be interesting to see how the results change when one of these dates as a surrogate for batch and adjusting using ComBat."

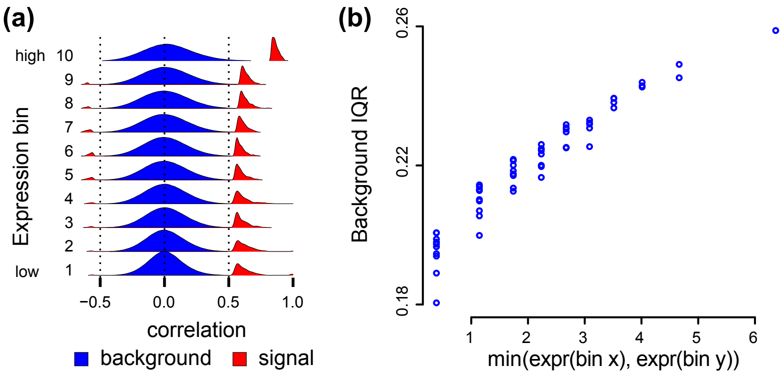

Figure 2. Variance stabilization does not remove the mean-correlation relationship. Same raw data as Figure 3a and 2c, but we apply a variance stabilizing transformation (as implemented by DESeq2) followed by removing 4 principal components. **a** Like Figure 3a, i.e. densities of the Pearson correlation between all genes within each of 10 expression bins (background) as well as the top 0.1%. **(b)** Like Figure 2c, i.e. the relationship between IQR of gene-gene correlation distribution and the lowest of the two expression bins associated with the submatrix.

Response: To directly address this question, we have used Combat with sample date as batch variable. Figure S14 shows that there is still a mean-correlation relationship. Furthermore, note that the correlations are not centered on zero. We interpret this as the batch effect not being fully removed. Finally, a short comment: note that Parsana et al. (2019) claims – unlike testing for differential expression – in a co-expression analysis, the top PCs are *always* associated with batch.

We now write in the section "The impact of removing principal components" in the Results: " *Many methods have been proposed to remove batch effects in differential expression analysis. To investigate the impact of alternatives to removing principal components, we use ComBat (Leek et al., 2012) to remove the effect of date of nucleic acid isolation batch in the expression matrix prior to constructing the correlation matrix. The correlation matrix exhibits the expected mean-correlation matrix (Supplementary Figure 3). Note the background distributions are not centered, which – based on the evaluations here – suggests that the is a remaining batch effect signal."*

> "3. This may be beyond the scope of the current manuscript, but I'd be curious to see the effect of considering sub-matrices of unequal size. For example, you could consider defining the size of the sub-matrices based on equal ranges of gene expression or equal within sub-matrix SDs."

Response: As our understanding, the reviewer is asking whether partitioning the correlation matrix using bins with equal range or SDs would have effect on the mean-correlation relationship and the downstream co-expression analysis.

Here we used the empirical distribution of the sub-matrix to approximate the true distribution of the correlations for genes inside the embedded sub-matrix. As we observed that

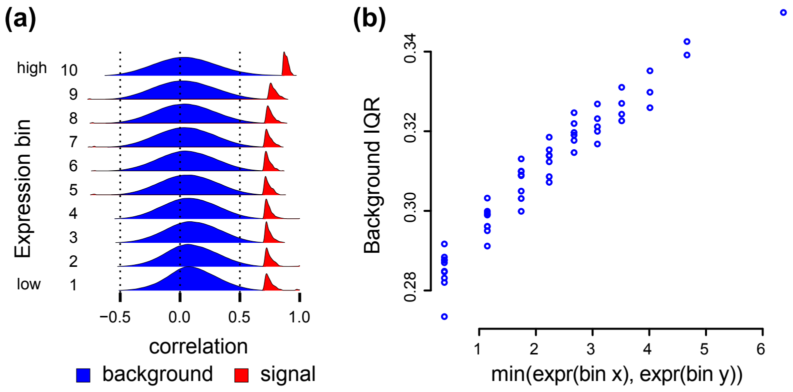

Figure 3. ComBat does not remove the mean-correlation relationship. Same raw data as Figure 3a and 2c. We apply ComBat (with the date of nucleic acid isolation as batch variable) prior to constructing the expression matrix, instead of removing principal components. **a** Like Figure 3a, i.e. densities of the Pearson correlation between all genes within each of 10 expression bins (background) as well as the top 0.1. **(b)** Like Figure 2c, i.e. the relationship between IQR of gene-gene correlation distribution and the lowest of the two expression bins associated with the submatrix.

the correlations' distribution is dependent on the expression level, controlling the within-sub-matrix ranges or SDs could to some degree control the within-sub-matrix range or variation of the correlations' distributions.

Here we used a bin size of 400 to make sure that the empirical distribution of the sub-matrix is approximately the same as the true distribution of the correlations between the genes within the bin. Defining bins based on the gene expression level or SDs could lead to bins with small size and an inaccurate approximation of the local distribution of correlations.

> "Minor Comments: 1. I found Fig 2b and 5b unintuitive. Is there possibly a better way to show this information?"

Response: Thank you for the suggestion. We have added color to the boxplots, colored by the value of IQR. This is a new display style where you need to follow the size of the boxes.

> "2. There appears to still be some widening of the background distributions and some shift in the signal distributions in Fig 5a, but it's hard to tell how much. I'd suggest adding vertical lines at -0.5, 0, and 0.5 to plots of this type."

Response: We have added vertical lines at -0.5, 0, and 0.5 to all the ridge plots.

> "3. I found the tissue-specific differences in background IQR (Fig 6a) interesting. Are these differences assumed to be purely technical?"

Response: We draw your attention to Supplementary Figure S5 where we make the same

plots, but instead of removing 4 PCs we use SVA to estimate the number of PCs (this removes 10-30 PCs per tissue, see Supplementary Table S1). In this supplemental figure, we see a stronger relationship between IQR and sample size compared with Figure 6b. Our conclusion to this is that a main driver of the background IQR are (tissue-specific) batch effects and sample size.

Because this result is part of our investigation on the impact of changing the number of PCs, it is described in section "The impact of removing principal components". We have added a callout to this in the paragraph where we describe Figure 6, where we now write

" *Below, we show that the relationship between sample size and IQR range becomes stronger when we remove a tissue-specific number of principal components."*

> "4. The general rarity of negatively correlated gene pairs (except in a few tissues) also perhaps warrants further discussion."

Response: This is also true before doing any kind of batch effect removal, which suggests that this rarity is not an artifact introduced by by batch effect removal. There is a study revealing evidence for that up-regulation effects are more efficient than down-regulation effects in fear. (Wiemer et al., 2021). We now draw attention to this in the Discussion where we write

" *In our examples we have focused on 9 tissues from GTEx. We find it noteworthy – and perhaps surprising – that almost all high correlations are positive. This happens both for unprocessed data, data where we have removed 4 (or more) principal components and data processed with SpQN."*

> "5. There are quite a few typos throughout the paper that should be fixed."

Response: We have run a better spell / grammar checker.

> "Summary: Overall, I think this is an important methodological advance in co-expression analysis and may open up other avenues of research. The paper would be strengthened by comparing to at least one (possibly naive) approach."

Response: Thank you; as you recommend we have considered a variance stabilizing transformation and alternatives to removing PCs for removing batch effects. While short, it is pretty clear these alternatives do not address the problem

Reviewer 2

(We have submitted a track changes document relative to the typesetting of our first submission. Additional text are marked with red. Deleted text are not marked.)

> "Wang et al. describe a computational method, "SpQN", for normalizing coexpression data to remove the previously reported bias such data tends to have in favor of highly-expressed genes. The authors demonstrate the method can effectively remove this bias on bulk data from GTEx as well as single cell RNA-seq data. The method and the demonstrations of its efficacy are well-described and an R package is provided (which I did not test).
>
> I believe this work has some merit, as the question for how to best exploit coexpression for biological discovery continues to be an important area of research. However, the authors give short shrift to the question of whether their approach is actually an improvement when it comes to biological inference. This may be difficult to convincingly address, but at least needs to be acknowledged by the authors and discussed as a limitation."

Response: We acknowledge that most of our evaluation is on the presence of bias and we have little focus on the important question of improving biological insight. In methods development it is usually much harder to show improved biological insight; this is of course the actual goal. However, co-expression analysis has particular challenges here, because it is not clear what co-expression **should** reflect. Unlike say a fold-change (differential expression) between conditions or cell-types, there is no real clear experimental way to validate co-expression. We believe most people think about "regulation" when they look at co-expression networks, but co-expression is not just about regulation.

Does this mean that co-expression is inherently flawed? Perhaps some people would think so, but we believe that co-expression – for all its flaws – sometimes enable insightful biological insights. The philosophy is not just theory, it has the real consequence that it is pretty hard to deal with the "improved biological insight", and we argue that most (all?) co-expression papers fall short here.

We have done some work to address the reviewer's comments (below), but there are clear limitations to our work. Ultimately, we have addressed this question by expanding our discussion of this important issue.

> "Specifically, while the authors have shown their method can reduce the mean-correlation bias, it seems possible it just introduces a new bias and needs to be evaluated. Will some truly biologically coexpressed gene pairs that are highly expressed be ignored after this normalization? Conversely, will some pairs of unrelated genes get inappropriately prioritized because they have low expression levels? As it stands, the manuscript primarily shows that the algorithm works at the task of removing the bias. But it is not clear that removing the bias

is desirable. The real challenge (which I don't expect to be met) is identifying lowly-expressed gene pairs that have been reproducibly and experimentally proven to be coexpressed or never coexpressed, and compare what happened to these pairs before and after the SpQN normalization."

Response: We think removing the bias is desirable in the sense that the "truth" should not be biased. However, we acknowledge this is different from **our approach** to removing the bias, which may very well have unintended consequences. And this later question is really the important question for our method.

We note – as above – that it is not clear what co-expression means from an experimental point of view.

"The only biologically motivated validation reported here is that transcription factors have more coexpression after the normalization. However, this seems a guaranteed outcome of the normalization, assuming TFs tend to be lowly expressed, and it seems hardly worth saying that more and better are not synonymous. Whether having more coexpression for these genes is actually a good or bad thing is not assessed. Doing so might not be very easy. In bulk tissue, coexpression is likely driven largely by cellular compositional variation. This is potentially addressed by the PC removal procedure, but in any case, showing that discovery of true direct TF-target relations in enhanced would be a much more convincing demonstration.

An obvious other tack is to look at the PPIs of Luck et al, which is already used in the manuscript. But for many reasons this would also be a weak validation. For one thing there is no strong reason to expect protein levels to be strongly correlated with RNA generally, but it could be better than nothing."

Response: We disagree that it is wrong to focus on transcription factors; they are very important regulatory genes. We agree that just assessing whether we get more (inferred) interactions involving transcription factors has issues.

To address this we have done the following. We have used the PPI database to define "true" transcription factor interactions (any interaction between a TF and another gene). This certainly has flaws, amongst them, the fact that PPI is not the same as co-expression. It also has the advantage that the "truth" is not biased by expression level. This results in 4, where we see that there is (perhaps, as expected) a big improvement in the true positive rate for lowly expressed genes and a drop in true positive rate for highly expressed genes, which comes out to a net total increase in true positives after SpQN. The increase/decrease is also expected – we use a fixed signal percentage to call co-expression (the x-axis in the plot), and this implies that if we increase the number of lowly expressed connections, we have to decrease the number of highly expressed connections because the percentage is fixed.

This analysis has several pitfalls including (1) using PPI to define "true" interactions (2)

using a fixed percentage to define ”signal”. Nevertheless it *suggests* that SpQN does not completely mess up total ”true” positives (although it clearly does drop ”true” positives from the highly expressed genes, but that is balanced by the increase amongst the lowly expressed genes). We acknowledge that this analysis is still far from a definitive answer to the question of ”improved insight into biology”, and we have highlighted this in our Discussion.

We now write in our Results section (note the two new supplemental figures are not included in this response):

” To quantify the impact of our method on transcription factor co-expression, we use a comprehensive list of 1,254 human transcription factors (Barrera et al., 2016). For each of our 9 exemplar tissues, we again threshold the correlation matrix and ask how many edges involve transcription factors with and without the use of SpQN. Figure 4a displays the percent increase in edges involving transcription factors following SpQN for various signal thresholds (ranging from 0.1% to 3%) of the correlation matrix for a single GTEx tissue (Additional tissues are depicted in Supplemental Figures S5, S6). This result shows an overall increase in edges involving transcription factors. Next, we computed the same percent change, but using protein-protein interactions involving transcription factors. We note that this is a flawed measure as protein-protein interactions are not the same as co-expression and because this analysis at best identifies co-factors and not downstream targets of the transcription factors. We observe an overall increase in edges involving transcription factors, but the increase – as expected – is for lowly expressed genes, whereas highly expressed genes show a decrease. This is partly explained by the zero-sum nature of calling edges based on a fixed percentage of interactions. We conclude that there is some evidence that SpQN improves the inference of interactions involving transcription factors, but this may come at the cost of decreased performance for highly expressed genes and that the overall performance depends on the expression distribution of the genes of interest.”

We now write in our Discussion section:

” Utilizing our method results in a greater number of connections involving transcription factors, an important class of regulatory genes. However, this increase may come at the expense of down prioritizing connections between highly expressed genes. The total benefits of SpQN on overall biological insight is likely to be impacted by the (unknown) expression distribution of the network of interest. For this reason, we suggest that users do not blindly apply the method.”

> ”In Crow 2016 and Farahbod 2019, the context was examining expression levels as an explanation for observed differences among the coexpression behavior of genes in different networks or different sets of genes within a network, not that the bias is an error in the construction of the networks that needs to be fixed. Presumably (but not demonstrated by the authors), SpQN would change the outcome of studies like Crow and Farahbod, but it is not clear whether the outcome would be desirable. For example, in Crow et al. it was observed that relative functional connectivity (as revealed by coexpression) of synaptic genes was partly explained by expression levels relative to other

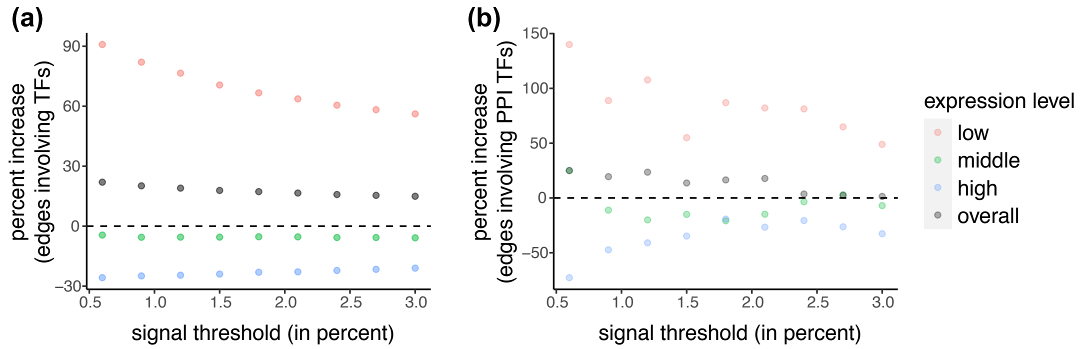

Figure 4. The impact of SpQN on transcription factor co-expression. Data is from adipose subcutaneous from GTEx. The percent increase in the number of edges (y-axis) identified after thresholding (x-axis) the correlation matrix. **(a)** Edges involving transcription factors. **(b)** Edges between genes with protein-protein interactions, where one of the involved genes is a transcription factor. Additional tissues are depicted in Supplemental Figures S5, S6.

> genes. Would removing the bias reduce that functional connectivity, or only obscure the fact that it is explained by expression levels? This seems worth discussion by the authors, if not explicit evaluation."

Response:

We thank the reviewer for this question. First, we agree that we did not demonstrate whether controlling for the expression level (e.g. applying SqQN) in Crow et al. (2016) and Farahbod and Pavlidis (2019) would be a desirable outcome. That being said, we want to highlight that Crow et al. (2016) note the expression level is the primary driver in their results:

" These results show that UMI-based networks contain functional information; however, the primary feature is their dependence on expression level."

and when the Crow et al. (2016) authors themselves control for expression level (e.g. restricting to only highly expressed genes), the author's conclusions admittedly change significantly resulting in a much smaller set of networks:

" To determine whether high expression is necessary for high performance, we restricted networks to only include high expressing genes (with median expression $\geq$ 16 *counts). This yielded much smaller networks, between 51 and 1368 genes in size (median = 227 genes), of which the synaptic set made up 30% (mean = 29.4% +/- 1.4 %). Synaptic gene set performance was greatly reduced in these networks (inset Fig. 4b, mean 0.49 +/- 0.03,* $p <$ 0.0002 *compared to non-thresholded networks, Wilcoxon rank sum test, n = 8)."*

To summarize, the authors note that controlling for the expression level does change their conclusions, but they also do not demonstrate whether it would be desirable. This rein-

forces the point we made earlier that it is difficult to determine whether a methods had ”improved biological insight” in the context of co-expression analysis, which most co-expression papers fall short of.

That being said, to address the reviewer’s question we have spent some time working with the data analyzed in Crow et al. (2016) and have found several problems with it. One issue is that one of the main datasets used in that paper (pre- and post-natal expression for ASD) has *very* small number of individuals (16 vs 18), compared to the studies in our manuscript (114-350 individuals per tissue), which brings into question the strength of *any* conclusions drawn. Furthermore, the analysis is unusual: instead of building a single (or two) networks across individuals, they build 34 individual specific network (utilizing they have 10-16 samples per individual) and present aggregations across networks. In short – after spending some time working on it – we do not think that a re-analysis of this data would give us strong biological validation of our method.

One evaluation criteria in the paper by Farahbod and Pavlidis (2019) is the reproducibility across datasets. However, as the gene expression level are expected to be highly reproducible across datasets (from the same tissue/context), correcting for gene expression level is unlikely to have an impact on reproducibility, as we note in our Discussion.

Ultimately, we believe the right thing is to control for expression level – and so does Crow et al. (2016) – but we acknowledge that we have little biological truth to back this up (as we also state throughout the reviewer’s other comments). However, in our opinion, anyone who wishes to ignore expression level needs to answer the converse question: if a result appears to be strongly associated with expression level, what is the evidence that the result is true?

> “If stronger validation is not possible, this work should be positioned as a proposal of an interesting computational method for normalizing correlation matrices (whatever their source, not necessarily biological) with respect to an external variable (whatever it is), consistent with the authors’ suggestion at the end of the discussion that this is a general method. As it stands, I could not be enthusiastic about recommending the method for use at this stage of the research, because there is no evidence that it actually improves the outcome of coexpression analysis from a biology standpoint. But it may spark discussion and further work in the community.”

Response: It is absolutely fair that no-one should apply SpQN blindly – especially so because it is a new approach. We have now stressed this in our Discussion where we write

“ Utilizing our method results in a greater number of connections involving transcription factors, an important class of regulatory genes. However, this increase may come at the expense of down prioritizing connections between highly expressed genes. The total benefits of SpQN on overall biological insight is likely to be impacted by the (unknown) expression distribution of the network

of interest. For this reason, we suggest that users do not blindly apply the method."

> "Minor:
>
> - The manuscript lacks a list of references. There is one in the preprint but it does not match the citations in the submitted manuscript, which I had to work around."

Response: We apologize. We do not know how that happened, but we have paid attention to this in our resubmission.

> "- Given the previous reports of this bias, the title and the way the work is presented seems to be a little bit of an overreach – both the title and the abstract makes it sound like the finding of the bias is original to this work. I'd suggest rewording to reflect that this work presents further documentation of the bias and a method for addressing it, not its discovery."

Response: We have changed the title and abstract..

Reviewer 3

(We have submitted a track changes document relative to the typesetting of our first submission. Additional text are marked with red. Deleted text are not marked.)

> "This paper introduced an interesting idea to investigate the mean-correlation relationship in bulk RNAseq and scRNAseq, how that affects the gene-gene correlation for example in pathway analysis. The authors also proposed a novel normalization method, spatial quantile normalization (SpQN). This paper proposed to provide further understanding beyond the previous publication (Freytag2015, in that another normalization RUV-random was suggested to correct the trend for correlation estimation).
>
> This draft has used a large gene expression dataset in GTEX as an data example. They also has used a scRNAseq dataset for demonstration.
>
> The effects on correlation calculation between previously removing PCs and their proposed method were investigated. That's interesting results to see.
>
> This draft is generally well written. However a few of the critical points need to be clarified so that the proposed method can be better understood/evaluated."

Response: Thank you for the nice comments.

> "Major comments:
>
> 1. Clarification of the bias due to the Mean-Correlation trend is needed.
>
> 1.1, In abstract, "This dependence introduces an unwanted technical bias in co-expression analysis". "Bias in Analysis" is a general statement. It may be fine in abstract. The precised definition of the "bias" needs to be defined early in the paper. Is it referring the bias of estimating the Pearson Correlation (e.g, the expected value of Pearson correlation)?"

Response: Thank you for highlighting the confusion about our use of the technical term "bias". We use the word bias in the following sense: that highly expressed genes are more likely to be highly correlated compared to lowly expressed genes. To link our use of bias to a classic statistical setting where an estimator is said to be biased if its mean is different from the population level parameter it claims to estimate, we need to consider network edges (ie. correlated genes) as estimators.

We are *not* referring to a bias in the estimation of individual correlations.

This is too complicated to state precisely in the abstract (although we give a one-sentence description). We now write the following in the section "The mean-correlation relationship biases co-expression analysis":

" Here we use the word "bias" to describe that highly correlated genes tend to be highly expressed. We are not using it to describe a potential bias of the empirical correlation estimator."

and in the Discussion section we write *" This is not a bias of the estimated Pearson correlation coefficient, but rather a preferential selection of highly expressed genes."*

> "Is the goal of SpQN to only adjust the estimated correlation? Does it output the adjusted gene expression?"

Response: SpQN will adjust the correlation matrix and the output of SpQN is an adjusted correlation matrix. We do not adjust the gene expression matrix. We have added a sentence which aims to clarify this to the Results:

" SpQN takes as input a correlation matrix and a gene-specific covariate (here: expression level) and outputs a normalized correlation matrix. The gene expression matrix is not modified."

> "1.2, On the other hand, there are alternative ways to describe the problem without using the word "Bias". The expression level may give some confidence in terms of the measurement error. Suppose we obtain correlations from two pairs, one from high expressed genes and the other very low expressed. The correlation calculated from the highly expressed pair would be more reliable than the other pair. Think of a pair with almost zeros. Expression level itself, to a certain extent, may serve a role as an effective sample size, or have some implications for the size of the error.
>
> Meanwhile, investigating the data expression of the high expressed genes that also have high correlation may be informative, since correlation itself has distribution assumption of linear relation. Invalid distribution may affect the statistical confidence of correlation.
>
> Picking highly correlated gene pairs, not based on a certain constant criterion but based on their statistical significance, could be meaningful, but it can to be clarified or better connect with the last equation in on page11 of Cor (Y1, Y2)"

Response: The reviewer is raising the issue that the pairwise correlation is likely to have less error (noise) for highly expressed genes than for lowly expressed genes, irrespective of any potential bias. A full analysis of this is difficult because it involves the 4th moment of the data (because we're looking at variance of a correlation). However, we claim that this should result in the background distributions being narrower (ie. less error) for highly expressed genes than for lowly expressed genes, which is the opposite of our observations.

We now write in "The distribution of gene-gene correlations depends on gene expression level" (Results): *" In addition to the behaviour of the true correlations, there is also the impact of measurement uncertainty. In general, higher expressed genes ought to have less noise when estimating their expression level and also their associated pairwise correlation, at least compared to lowly expressed genes. This suggests that measurement noise ought to be decreasing as expression*

level increases, the opposite trend of what we observe."

> "2. Clarification of the trend of Mean-Correlation is needed. The "mean-correlation relationship" seems to be defined in two different ways throughout the paper. Which of the two has been claimed in the paper needs to be further clarified.
>
> The two definitions seemly are, (1) In Abstract, "higher correlation is more likely to happen among highly expressed genes." That suggests, mean estimated correlation conditional on the mean expression $E[Cor(X,Y) \mid min(E(X),E(Y))]$ This is also implied from the negative-binomial-based motivation on page 3.
>
> (2) In the 2nd paragraph of Page 4, "This model explains why the width of the background distributions decrease with decreasing expression level (the adjustment factors decreases) and suggests that the "true" width of the background distribution is observable for highly expressed genes. "
>
> If I understand correctly, that (2) suggests, variance (or distribution) of estimated correlation conditional on the mean expression $var[Cor(X,Y)) \mid min(E(X),E(Y))]$.
>
> If higher dispersion is to be claimed, comparing the standard error (or the variance) of the estimated cor(Y1,Y2) and of the estimated cor(Z1, Z2) may better represent the idea."

Response: This question (together with the other clarifying questions above) has highlighted important conceptual gaps in our thinking about our (previously) proposed explanation for **why** this happens.

First: we believe we are quite consistent in using the term mean-correlation relationship: it refers to the fact that the width of the background distributions depends on the expression level. What we were unclear about is why this happens. In our graphical display we plot a distribution across pairs of genes. This distribution will be affected by the variance of the estimator (the formula in (2)), by systematic distortions of the expected correlation and by other things.

Our theory section establishes the following. Say we have two populations of genes. One population is highly expressed and the other one is lowly expressed and the correlation distribution of the true unobserved expression (Z in our notation) is *the same* in the two populations. Then the observed counts (Y in our notation) will have the same correlation as the Zs in the highly expressed groups, but be systematic lower in the lowly expressed group. This ignores changes in the variance of the correlation estimator (as commented on above) but can explain why the width of the distribution is smaller for the lowly expressed group. Note that this result is not so easy to write with a formula along the lines of the reviewer's comment, because it is a distribution across gene pairs. However, this result only holds when the true unknown correlation is non-zero. And we tend to believe that the true correlation matrix is sparse (ie. most genes are uncorrelated). That is

an assumption, but we like that assumption. In conclusion, our theory is an unsatisfying answer to the question of why?

We now write in "A model-based investigation of the mean-correlation relationship in RNA-seq data" (Results): " *This model explains why – for genes with a true non-zero correlation – the width of the background distributions decrease with decreasing expression level (the adjustment factors decreases) and suggests that the "true" width of the background distribution is observable for highly expressed genes. Furthermore, it suggests that the background distributions in different submatrices are roughly related through a scaling transformation. However, this argument falls apart if we believe the true expression network to be sparse, ie. that most genes are truely uncorrelated."* and conclude " *In summary, this model is at best a partial explanation of the observed phenomena."*

> "3. Both the chosen bulk RNAseq and single cell RNAseq data have larger n (large mathematical sample sizes). Since PCA and SVA type of methods mostly were suggested in the large bulk RNAseq data like GTEX, the performance of SpQN in small sample sizes will be also interesting to know. It's possible if n=3 per sample group is too small for a good correlation calculation. If there are large n (say 5 or 10) from well-controlled and well-designed experiments (e.g., mouse in wet lab) will be useful for this question."

Response: Previous studies have suggested that a sample size of at least 20 is required for co-expression network analysis (Ballouz, Verleyen, and Gillis, 2015; Langfelder and Horvath, 2008). In our opinion, this bound is very low (ie. we would be vary of doing co-expression with this few samples), so we have not pursued this question further.

Related, We have included an analysis of a dataset with 30 samples in drosophila showing the mean-correlation relationship in a small sample size setting (but not demonstrating the impact of SpQN). This dataset is not an apple-to-apple comparison of 300 to 30 samples (since the drosophila samples are part of a time course experiment).

> "Minor comments:
>
> 1. Figure 2b, the 2D box plot is informative but is hard to read. Adding colors or density scale would be beneficial."

Response: We have added color to the 2D box plot, colored by the IQR value.

> "2. On page 5, in constructing the empirical distribution using the enclosing submatrices, how it is obtained at a boundary was not explained. The submatrices at boundaries, unlike the usual submatrices inside, are asymmetric and may cause bias. In other words, the F_{emp} at the bottom left is constructed using higher-expressed gene pairs and that at the top right will be constructed using lower-expressed gene pairs."

Response: The entries at the boundary of the correlation matrix were assigned to the submatrices in the following way, now described in the Methods section:

" *The set of submatrices* $\{X_{i,j}\}$ *is assigned to be disjoint and same-distance bins, with distance equals to that of* $Y_{i,j}$, *written as*

$$X_{i,j} = \{\{g, g'\} : n_1(i) < g \leq n_2(i),\, n_1(j) < g' \leq n_2(j)\},$$
$$i, j = 1, 2, ..., n_{group}, https : //www.overleaf.com/project/5c904c36dc13092528f03c76$$

where

$$n_1(x) = \begin{cases} 0, & \text{if } x = 1 \\ n_2(x-1), & \text{otherwise} \end{cases}$$
$$n_2(x) = \begin{cases} d/2 + w/2, & \text{if } x = 1 \\ n_{gene}, & \text{if } x = n_{group} \\ n_1(x) + d, & \text{otherwise.} \end{cases}$$

"

> "3. On page 5, the authors mention "reference distribution" for the first time: "The choice of reference distribution. . . ." It seems they refer it to "the target distribution" in the quantile function definition. Need to unify the terminology."

Response: We used the two terms interchangeably. To minimize confusion, we have standardized on "target distribution" and have changed all instances of "reference" to "target".

> "4, A vertical line at x=0 for the blue peaks in Figure 2a will be helpful to see the distribution of correlation. The same suggestion also applies to other later blue peaks of correlations in figures (3a, 5a, 10a, 10c)."

Response: We have added vertical lines at -0.5, 0, 0.5 to the ridge plots.

Reviewer checklist

> "Have the authors made all data and (if applicable) computational code underlying the findings in their manuscript fully available? The PLOS Data policy requires authors to make all data and code underlying the findings described in their manuscript fully available without restriction, with rare exception (please refer to the Data Availability Statement in the manuscript PDF file). The data and code should be provided as part of the manuscript or its supporting information, or deposited to a public repository. For example, in addition to summary statistics, the data points behind means, medians and variance measures should be available. If there are restrictions on publicly sharing data or code —e.g. participant privacy or use of data from a third party—those must be specified.
>
> Reviewer #1: Yes
>
> Reviewer #2: No: There is a github repository with the code for the method, but I don't see the data used or code for analysis of the data presented in the manuscript. At least, it is not obvious as there is no documentation pointing to where they are.
>
> Reviewer #3: No: The develop R package is publicly available in Github. I reckon the authors will make the analysis code available when paper is accepted."

Response: We had – by mistake – not included a link to our analysis code, only to our software package (which does contain our method, arguably the most important product.

- The package is available at `https://www.bioconductor.org/packages/spqn`.
- The paper code is available at `https://www.github.com/hansenlab/spqn_paper`.
- Some data cleaning code (GTEx) is available in `https://www.bioconductor.org/packages/spqnData`.

This is now listed in the new "Data Availability" section formatted according to PLOS guidelines.
