# Supplementary material for: Addressing the mean-correlation relationship in co-expression analysis
Source: PLoS Comput Biol. 2022 Mar 30;18(3):e1009954. doi: 10.1371/journal.pcbi.1009954 (PMC9009771; doi:10.1371/journal.pcbi.1009954)
Supplement: Attachment — Submitted filename: v2 reviewPlosComp July 2021 DW_CHO.docx [file pcbi.1009954.s002.docx]

This paper introduced an interesting idea to investigate the mean-correlation relationship in bulk RNAseq and scRNAseq, how that affects the gene-gene correlation for example in pathway analysis. The authors also proposed a novel normalization method, spatial quantile normalization (SpQN).

This paper proposed to provide further understanding beyond the previous publication (**Freytag2015**, in that another normalization RUV-random was suggested to correct the trend for correlation estimation).

This draft has used a large gene expression dataset in GTEX as an data example. They also has used a scRNAseq dataset for demonstration.

The effects on correlation calculation between previously removing PCs and their proposed method were investigated. That's interesting results to see.

This draft is generally well written. However a few of the critical points need to be clarified so that the proposed method can be better understood/evaluated.

Major comments:

1. Clarification of the bias due to the Mean-Correlation trend is needed.

1.1, In abstract, "This dependence introduces an unwanted technical bias in co-expression analysis".

"Bias in Analysis" is a general statement. It may be fine in abstract. The précised definition of the "bias" needs to be defined early in the paper. Is it referring the bias of estimating the Pearson Correlation (e.g, the expected value of Pearson correlation)?

Is the goal of SpQN to only adjust the estimated correlation? Does it output the adjusted gene expression?

1.2, On the other hand, there are alternative ways to describe the problem without using the word "Bias". The expression level may give some confidence in terms of the measurement error. Suppose we obtain correlations from two pairs, one from high expressed genes and the other very low expressed. The correlation calculated from the highly expressed pair would be more reliable than the other pair. Think of a pair with almost zeros. Expression level itself, to a certain extent, may serve a role as an effective sample size, or have some implications for the size of the error.

Meanwhile, investigating the data expression of the high expressed genes that also have high correlation may be informative, since correlation itself has distribution assumption of linear relation. Invalid distribution may affect the statistical confidence of correlation.

Picking highly correlated gene pairs, not based on a certain constant criterion but based on their statistical significance, could be meaningful, but it can to be clarified or better connect with the last equation in on page11 of Cor (Y1, Y2).

2. Clarification of the trend of Mean-Correlation is needed.

The “mean-correlation relationship” seems to be defined in two different ways throughout the paper. Which of the two has been claimed in the paper needs to be further clarified.

The two definitions seemly are,

(1) In Abstract, "higher correlation is more likely to happen among highly expressed genes.” That suggests, mean estimated correlation conditional on the mean expression E[Cor(X, Y) | min(E(X), E(Y))]

This is also implied from the negative-binomial-based motivation on page 3.

(2) In the 2nd paragraph of Page 4,

"This model explains why the width of the background distributions decrease with decreasing expression level (the adjustment factors decreases) and suggests that the “true” width of the background distribution is observable for highly expressed genes. "

If I understand correctly, that (2) suggests, variance (or distribution) of estimated correlation conditional on the mean expression var[Cor(X, Y)) | min(E(X), E(Y))].

If higher dispersion is to be claimed, comparing the standard error (or the variance) of the estimated cor(Y1,Y2) and of the estimated cor(Z1, Z2) may better represent the idea.

3. Both the chosen bulk RNAseq and single cell RNAseq data have larger n (large mathematical sample sizes).

Since PCA and SVA type of methods mostly were suggested in the large bulk RNAseq data like GTEX, the performance of SpQN in small sample sizes will be also interesting to know. It's possible if n=3 per sample group is too small for a good correlation calculation. If there are large n (say 5 or 10) from well-controlled and well-designed experiments (e.g., mouse in wet lab) will be useful for this question.

Minor comments:

1. Figure 2b, the 2D box plot is informative but is hard to read. Adding colors or density scale would be beneficial.

2. On page 5, in constructing the empirical distribution using the enclosing submatrices, how it is obtained at a boundary was not explained. The submatrices at boundaries, unlike the usual submatrices inside, are asymmetric and may cause bias. In other words, the F_emp at the bottom left is constructed using higher-expressed gene pairs and that at the top right will be constructed using lower-expressed gene pairs.

3. On page 5, the authors mention “reference distribution” for the first time: “The choice of reference distribution….” It seems they refer it to “the target distribution” in the quantile function definition. Need to unify the terminology.

4, A vertical line at x=0 for the blue peaks in Figure 2a will be helpful to see the distribution of correlation. The same suggestion also applies to other later blue peaks of correlations in figures (3a, 5a, 10a, 10c).
